# Effect of Filtered Back-Projection Filters to Low-Contrast Object Imaging in Ultra-High-Resolution (UHR) Cone-Beam Computed Tomography (CBCT)

**DOI:** 10.3390/s20226416

**Published:** 2020-11-10

**Authors:** Sunghoon Choi, Chang-Woo Seo, Bo Kyung Cha

**Affiliations:** 1 Safety Measurement Institute, Korea Research Institute of Standards and Science (KRISS), Daejeon 34113, Korea; reschoi127@gmail.com; 2 Department of Radiation Convergence Engineering, Yonsei University, Wonju 26493, Korea; cwseo@yonsei.ac.kr; 3 Electro-Medical Device Research Center, Korea Electrotechnology Research Institute, Ansan 15588, Korea

**Keywords:** ultra-high resolution, cone-beam computed tomography, low-contrast object, optimal filter, modulation transfer function, noise power spectrum

## Abstract

In this study, the effect of filter schemes on several low-contrast materials was compared using standard and ultra-high-resolution (UHR) cone-beam computed tomography (CBCT) imaging. The performance of the UHR-CBCT was quantified by measuring the modulation transfer function (MTF) and the noise power spectrum (NPS). The MTF was measured at the radial location around the cylindrical phantom, whereas the NPS was measured in the eight different homogeneous regions of interest. Six different filter schemes were designed and implemented in the CT sinogram from each imaging configuration. The experimental results indicated that the filter with smaller smoothing window preserved the MTF up to the highest spatial frequency, but larger NPS. In addition, the UHR imaging protocol provided 1.77 times better spatial resolution than the standard acquisition by comparing the specific spatial frequency (*f*_50_) under the same conditions. The *f*_50_s with the flat-top window in UHR mode was 1.86, 0.94, 2.52, 2.05, and 1.86 lp/mm for Polyethylene (Material 1, M1), Polystyrene (M2), Nylon (M3), Acrylic (M4), and Polycarbonate (M5), respectively. The smoothing window in the UHR protocol showed a clearer performance in the MTF according to the low-contrast objects, showing agreement with the relative contrast of materials in order of M3, M4, M1, M5, and M2. In conclusion, although the UHR-CBCT showed the disadvantages of acquisition time and radiation dose, it could provide greater spatial resolution with smaller noise property compared to standard imaging; moreover, the optimal window function should be considered in advance for the best UHR performance.

## 1. Introduction

Ultra-high-resolution (UHR) computed tomography (CT) has been used in commercial applications since 2017 due to it features of higher image spatial resolution [1]. Kakinuma et al. used a prototype UHR-CT that was operated with 0.25 mm detector pixel size and 0.1 mm reconstructed image pixel interval at 0.25 mm slice thickness [2]. Another experiment with a clinical UHR-CT scanner (Aquilion Precision, Canon Medical Systems) reported that the system was operated in the same condition of that in the previous paper [3]. The clinical UHR-CT has three scan modes: normal, high-resolution (HR), and super-high-resolution (SHR) modes, which support 512 × 512, 1024 × 1024, and 2048 × 2048 image matrixes, respectively, in a given reconstruction field of view (FOV) [4]. Judging from the previously published papers, UHR-CT generally can be distinguished from conventional high-resolution CT (CHR-CT) because it makes use of pixel sizes below 0.25 mm at image matrixes above 512. It has been reported that CHR-CT uses pixel sizes ranging from 0.23 mm [5] to 0.35 mm [6]. The clinical aspects of UHR-CT include reduction of vascular continuity of the coronary arteries, visualization of fine structures of lungs, such as peripheral pulmonary vessels less than 1 mm in size, and artifact reduction such as blooming [7,8].

Recent X-ray detector technology in both multi-row and the flat-panel detectors (FPDs) enables high-resolution acquisition at a small pixel size of less than 0.25 mm [9]. Following these efforts, dedicated UHR cone-beam CT (CBCT), e.g., the OnSight 3D system (Carestream Healthcare, Rochester, NY, USA), has been introduced for extremity scans at lower cost and radiation doses compared to multi-detector CT (MDCT) systems [10]. The OnSight 3D system are mounted with a CsI:Tl scintillator-based complementary metal-oxide semiconductor (CMOS) FPD with a pixel size of 139 µm. UHR-CBCT can ultimately improve the visualization of bone morphometry and contribute to the diagnosis of osteoporosis and osteoarthritis, and detection of fine fractures, which typically require measurements in the range of 0.05–0.2 mm [11]. In general, the FPD could be operated in detector pixel binning mode, which is the process of combining the adjacent electric charges into one pixel [12]. This can reduce both the electronic and quantum noise, and decrease the image readout time at a higher frame rate. The user selects the FPD operation in either full or binning mode, which can optimally satisfy the need of correlation between the resolution and frame rate. 

However, the image at higher resolution is not always good, especially for low-contrast detection tasks due to the enhanced noise level during the process of filtered back-projection (FBP) image reconstruction [13]. The “low contrast” of the image can be described as low discrimination between the target and background. The spatial resolution measurement in high-density materials, such as bar pattern and tungsten wire, is an easy task for both standard and UHR CT imaging. However, medical image quality of low-contrast objects is defined in terms of how well the tradeoff relationship between the resolution and noise is obtained from the image [14]. The amount of noise suppression at high frequencies is adjustable by setting either different cutoff frequency levels or different smoothing functions implemented on the CT sinogram. The higher the cutoff frequency level, the sharper but noisier the reconstructed image [15]. This, in turn, results in reconstructed image quality, thereby greatly influencing the detectability of objects by human observers [16]. Unfortunately, choosing an optimal filter scheme relies on experience, because there is no global function that can accept all principal signals underlying the entire frequency range. Therefore, the effect of the reconstruction filters on different materials in UHR-CBCT should be studied to provide useful information when observing a tiny amount of information during UHR acquisition.

In this study, we measured the spatial resolution of five different cylindrical objects according to four different UHR acquisition modes using six different filter schemes. The self-developed UHR-CBCT system, which is installed at the authors’ institution (Korea Electrotechnology Research Institute, Ansan, Korea) was used for acquiring the CBCT images in both standard and high-resolution modes. This study aimed to evaluate the effect of filter schemes on the spatial resolution that underlies each imaging object and to suggest the optimal filter scheme in UHR-CBCT depending on the different object materials. 

## 2. Materials and Methods

### 2.1. Ultra-High-Resolution Cone-Beam Computed Tomography System and Imaging Configurations

**Figure 1:**
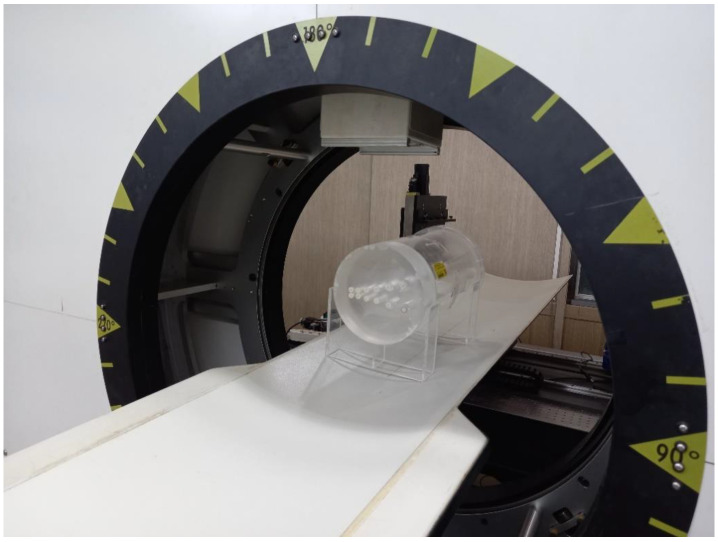
Photograph of the prototype cone-beam computed tomography (CBCT) system capable of both standard and ultra-high resolution (UHR) acquisition.

A photograph and a specification of a prototype CBCT system are provided in Figure 1 and Table 1. Our system was mounted with an amorphous silicon (aSi)-based thin-film transistor (TFT) array FPD (PaxScan 4030CB, Varian Imaging Products, Palo Alto, CA) and was operated in full and binning acquisition modes. As shown in Table 2, the imaging configuration was categorized into four subsections according to the two acquisition resolution setups and two reconstructed image resolutions. Each configuration was named depending on the row and column number of the matrix. 

The center of rotation of the system was registered using the calibration phantom while rotating a full 360° with a 1° angle step for projection view image acquisition of 361 images. The 0.25 and 0.5 mm slice thicknesses were chosen based on previous studies [2,3,4]. The readout time of FPD with a 2 × 2 binning mode acquisition was four times faster than that of a full mode acquisition; therefore, a lower total acquisition time and lower radiation exposure were achievable owing to the higher framerate in the binning mode. All FBP reconstruction algorithms were self-programmed and coded in C++ with the CUDA toolkit version 10.0 using a single GPU card (GTX Titan-Xp, NVIDIA Co., Ltd., Santa Clara, CA, USA).

### 2.2. CT Performance Phantom

We used the CIRS Model 610 American Association of Physicists in Medicine (AAPM) CT performance phantom to measure spatial resolution and noise property. The CT number linearity insert (Part No. 610-02), which includes five cylinders with different densities, was a targeted imaging object for resolution measurement. The detailed specifications of the inserted cylinders are given in Table 3. Each cylinder has the same size and shape and has a low contrast against the background material, thus presenting a small absolute signal difference between the two materials. The larger the material index, the smaller the difference between the background and target material densities. Note that a small absolute difference between the densities of two materials does not always guarantee a small image contrast because the CT numbers are represented by the linear attenuation coefficients which are dependent on both X-ray energy and density.

The insert (Part No. 610-01-05) is comprised of a uniform material with an aluminum pin at the center, and is a good candidate for measuring noise power. We assumed that the noise behaviors were the same for all materials because quantum and electronic noise, which are both stochastic events, are dominant over the entire area.

### 2.3. Ramp Filter Design in Spatial Domain and Six Different Window Functions

Linear filtering can be categorized into two methods: applying the convolution kernel in the spatial domain and linear multiplication of a transfer function in the Fourier domain. A band-limited ramp filter constructed in the Fourier domain is defined as follows:

(1) $$RAMP{\left(\omega \right)}_{A}=\{\begin{array}{c} \left|\omega \right|,\\ 0, \end{array}\;\begin{array}{c} \mathrm{if}\;\left|\omega \right|\leq 0.5\;\mathrm{lp}/\mathrm{mm}\\ \mathrm{otherwise} \end{array}$$

where *ω* is the discretized spatial frequency by considering the Nyquist frequency. However, the ramp filter in Equation (1) has a zero at ω=0 lp/mm such that the signals at the DC offset (zero frequency component) after linear multiplication go to zero. The Fourier transform of the ramp convolution kernel constructed in the spatial domain can be defined as follows [17]:

(2) $$RAMP{\left(\omega \right)}_{B}=FT\left\{ramp\left(n\right)\right\}=\int_{-\infty }^{\infty }ramp\left(n\right)e^{-i2\pi \omega }d\omega$$

(3) $$ramp\left(n\right)=\{\begin{array}{c} 1/4,\\ 0,\\ -1/{\left(n\pi \right)}^{2}, \end{array}\;\begin{array}{c} \mathrm{if}\;n=0\\ \mathrm{if}\;n\;\mathrm{is}\;\mathrm{an}\;\mathrm{even}\;\mathrm{number}\\ \mathrm{if}\;n\;\mathrm{is}\;\mathrm{an}\;\mathrm{odd}\;\mathrm{number} \end{array}$$

**Figure 2:**
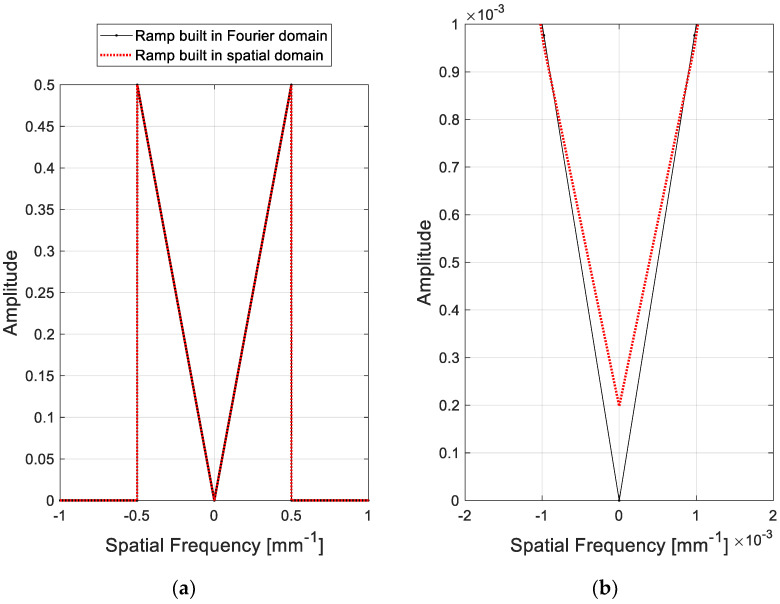
(**a**) Comparison of the ramp filters designed in different domains and (**b**) its magnified plot near the DC (zero frequency) component.

The ramp filter in Equation (2) does not include a zero, as shown in the comparison of the two shapes in Figure 2. Filtering with non-zero conditions avoids the zero signals that might have occurred if the filters were used with zero conditions.

Many window functions have been introduced depending on the strength of noise suppression at different cutoff frequencies for each purpose [18]. However, the reduction of the critical signal is inevitable during noise suppression; therefore, the optimal window function is often heuristically chosen after multiple reconstruction trials. Six different smoothing windows were implemented herein in the ramp filter. Each window function was followed by the equation summarized in Table 4, where a term *L* in (b), (c), (d), and (f) indicates the length of the window.

### 2.4. Modulation Transfer Function (MTF)

**Figure 3:**
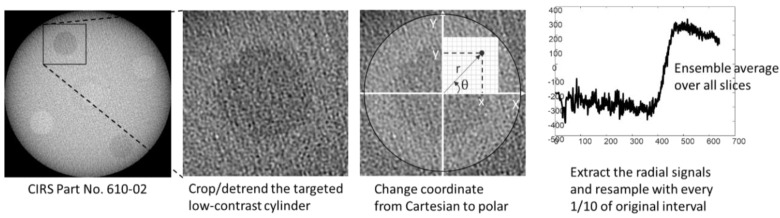
A depicted workflow for 1D edge spread function (ESF) measurement of the targeted low-contrast material.

Spatial resolution for each imaging configuration and each filter scheme was evaluated by the MTF measurement of the cylindrical materials as conducted by Richard et al. [19]. After subtracting the two-dimensional planar fit from the original region of interest (ROI) of each targeted cylinder, the radial pixel values around the edge of the circular shape were rearranged to yield a one-dimensional edge spread function (ESF). When converting the image grid from a Cartesian to polar map, the center of each disk was measured on a binary image through a gray-level threshold. The ESF, which is equivalent to the radial profile of the circle, was resampled with one-tenth of the reconstructed pixel size to reduce the non-uniformly distributed pixel noise [20]. The final ESF was derived by averaging the ESFs measured from consecutive axial slices. The MTF was the Fourier amplitude of the derivative of the ensemble-averaged ESF. In addition, the high-frequency noise of the ESF derivative was relieved through a Hanning window having the same length as the ESF size. The overall process of radial MTF measurement is depicted in Figure 3.

### 2.5. Normalized Noise Power Spectrum

**Figure 4:**
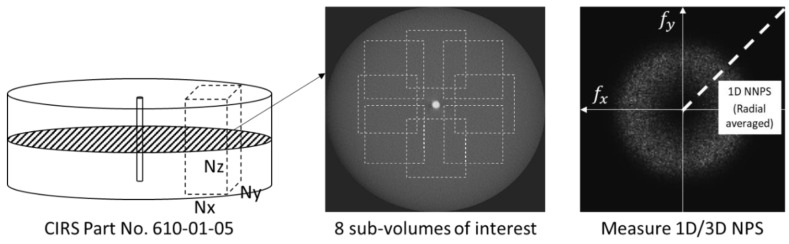
A schematic illustration for deriving 3D NPS.

The normalized noise power spectrum (NNPS) was measured to quantify the noise level in the homogeneous volume of interest (VOI) of the poly methyl methacrylate (PMMA) background. The three-dimensional (3D) NPS was measured as described in Figure 4. The eight different VOIs without interference of any structure with the size of 150 × 150 × 45 (300 × 300 × 90 for high-resolution reconstruction) were selected for measuring the 3D NPS. Each sub-volume overlapped with others to evaluate the radially and symmetrically distributed noise property (location independent noise pattern) [21]. 

Each mean subtracted sub-volume patch was Fourier transformed, absolute squared, and ensemble averaged to yield the power spectrum as follows [22]:

(4) $$NPS\left(f_{x},\;f_{y},\;f_{z}\right)=\frac{1}{2}\frac{d_{x}d_{y}d_{z}}{N_{x}N_{y}N_{z}}\langle {\left|ℱ\left[S\left(i,j,k\right)-\bar{S}\right]\right|}^{2}\rangle ,$$

where $f_{x}$, $f_{y}$, and $f_{z}$ are spatial frequencies (mm^−1^), $d_{x}$, $d_{y}$, and $d_{z}$ are pixel sizes (mm), $N_{x}$, $N_{y}$, and $N_{z}$ are the numbers of voxels in the sub-volume patch, ℱ[·] is the fast Fourier transform operator, and S(i,j,k) and $\bar{S}$ indicate each voxel value and the mean intensity of the sub-volume patch, respectively. The 1D NNPS can be derived by radially averaging the 3D NPS [23].

## 3. Results

### 3.1. Filter Shape

**Figure 5:**
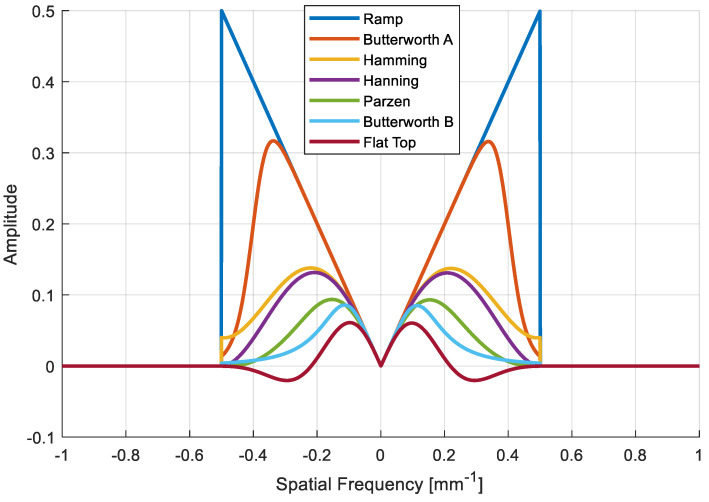
Different band-limited filter shapes as a function of frequency response.

Six different Fourier transformed and band-limited filters designed in the spatial domain with regard to the frequency response are depicted in Figure 5. Because the Fourier transformed sinograms were forced to be band limited with a band width of 0.5, the signals outside of the band frequency range went to zero, as shown in Figure 5. Similarly, each window function was also band limited and multiplied by the band-limited ramp filter. The magnitude of the filter at high frequencies was rejected when going from scheme (a) to (f) in Table 4, which is generally interpreted as noise suppression. Unlike other filters, some of the value of the flat-top window are negative.

### 3.2. Reconstructed Images with Different Filters and Configurations

**Figure 6:**
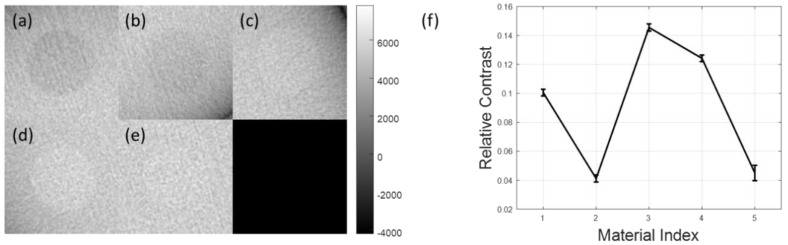
Reconstructed image with configuration (1, 1) using the Hanning window. (**a**–**e**) The cropped region of interest (ROI) around the center of each material, and (**f**) relative contrast between the target and background with standard deviation error bar as a function of each material.

Figure 6 shows the reconstructed images with configuration (1, 1) using the Hanning window and its cropped ROI images around the centers of five different materials. The relative contrast between each material and background with standard deviation error are plotted in Figure 6f. All five materials showed a low contrast, showing a small relative contrast below 0.15 (maximum contrast is 1). As mentioned above, the higher density material does not always represent the higher CT number when we measure the contrast between each material and the background (PMMA). M2 and M5 showed the lowest contrast among the five materials. 

**Figure 7:**
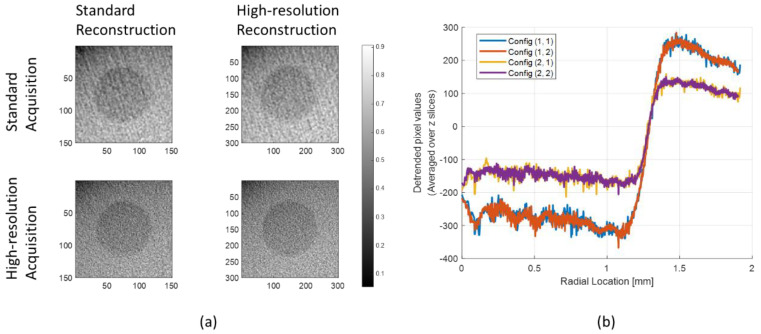
(**a**) Reconstructed image with different configurations using the Hanning window and (**b**) the radial profile of each configuration.

The reconstructed cropped images of M1 with different configurations using the Hanning window are shown in Figure 7a. Figure 7b shows the radial profiles of each image grid in Figure 7a. The images reconstructed using standard detector resolution (configuration (1, 1) and (1, 2)) showed an unstable fluctuation in their radial profiles at the initial radial location. On the contrary, the images of configurations (2, 1) and (2, 2) showed relatively flat signals.

**Figure 8:**
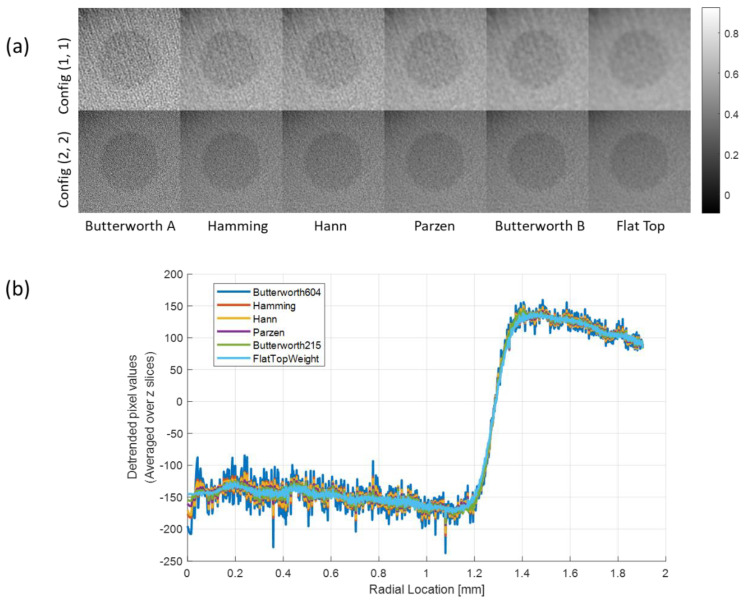
(**a**) Reconstructed image of M1 with different configurations according to the different windows and (**b**) the radial profile of configuration (2, 2).

**Figure 9:**
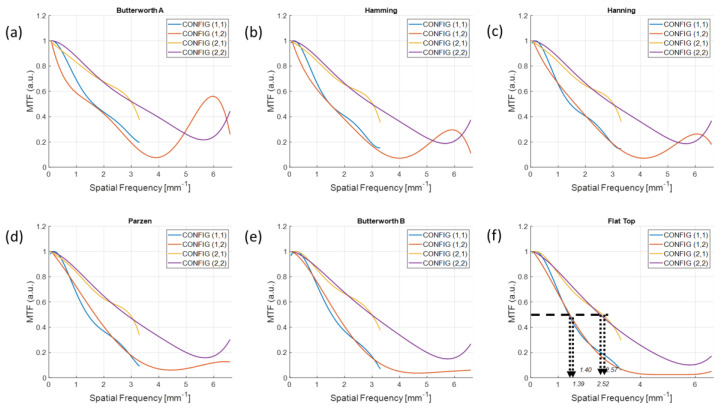
Modulation transfer functions (MTFs) for different filter schemes from the (**a**) Butterworth A, (**b**) Hanning, (**c**) Hamming, (**d**), Parzen, (**e**) Butterworth B, and (**f**) Flat Top windows with different configurations. The *f*_50_s measured in the images implemented with the flat top window were 1.39, 1.40, 2.52, and 2.57 lp/mm for the configuration (1, 1), (1, 2), (2, 1), and (2, 2), respectively.

To understand the effect of filter schemes on image quality, the reconstructed images of M1 with different filters using configurations (1, 1) and (2, 2) are shown in Figure 8a. The radial profiles in Figure 8b correspond to the bottom row images in Figure 8a (configuration (2, 2)). The fluctuations of the radial profiles are gradually smoothed with an increase in the index number of filter schemes, demonstrating that the high-frequency noise was rejected by using the smoothing windows. The more oscillations in the signal, the coarser the MTF curve, as shown in Figure 9a.

### 3.3. Modulation Transfer Function

Six different MTFs for each filter scheme measured in the reconstructed images of M1 are shown in Figure 9. The higher the resolution of the reconstructed images, the better the MTF is preserved up to the high frequencies. In Figure 9f, *f*_50_, which indicates the specific spatial frequency when the MTF is dropped to 0.5, was 1.39, 1.40, 2.52, and 2.57 lp/mm for the configurations (1, 1), (1, 2), (2, 1), and (2, 2), respectively. The effect of detector resolution on the reconstruction image resolution was minor when we compared the curves between configurations (1, 1) and (1, 2) (or (2, 1) and (2, 2)).

**Figure 10:**
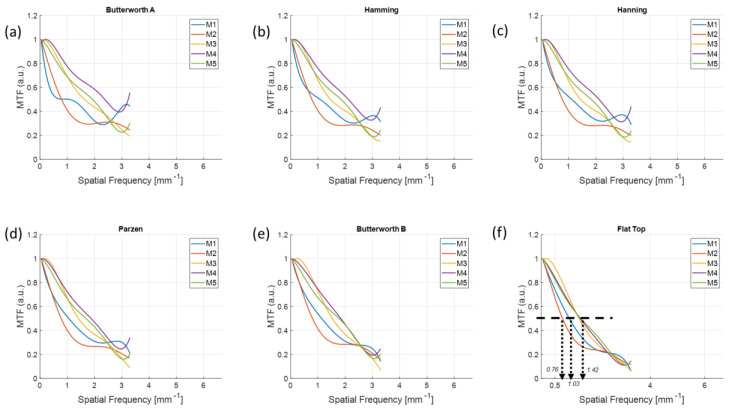
MTFs for different materials with the configurations (1, 1) using different filter schemes from the (**a**) Butterworth A, (**b**) Hanning, (**c**) Hamming, (**d**), Parzen, (**e**) Butterworth B, and (**f**) Flat Top windows. The orders of *f*_50_s as a function of different materials were different for each filter scheme.

**Figure 11:**
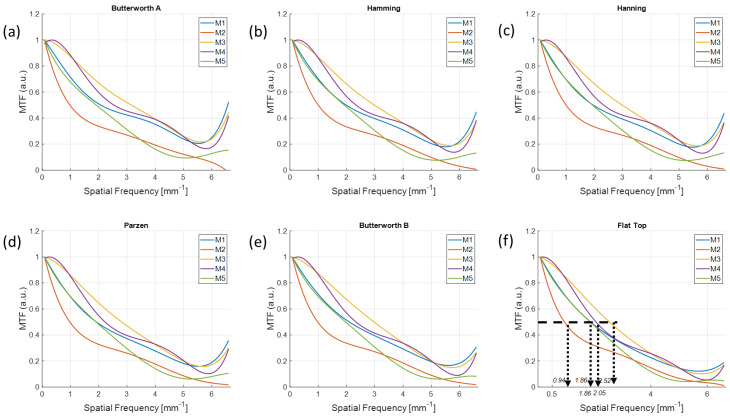
MTFs for different materials with the configurations (2, 2) using different filter schemes from the (**a**) Butterworth A, (**b**) Hanning, (**c**) Hamming, (**d**), Parzen, (**e**) Butterworth B, and (**f**) Flat Top windows. The order of *f*_50_s as a function of different materials was M3, M4, M1, M5, and M2.

The MTF curves measured in the reconstructed images of each material using configurations (1, 1) and (2, 2) are shown in Figure 10 and Figure 11. As shown in Figure 11f, the *f*_50_s were 0.94, 1.86, 2.05, and 2.52 and 1.86 lp/mm from M1 to M5, respectively, which demonstrates that MTFs were preserved up to high frequencies of the order of M3, M4, M1, M5, and M2; that is, in the order of the relative contrast in Figure 6f. In contrast, the imaging configuration (1, 1) not only did not follow the order of contrast, but also presented different orders of *f*_50_s for the different filter schemes.

### 3.4. Normalized Noise Power Spectrum

**Figure 12:**
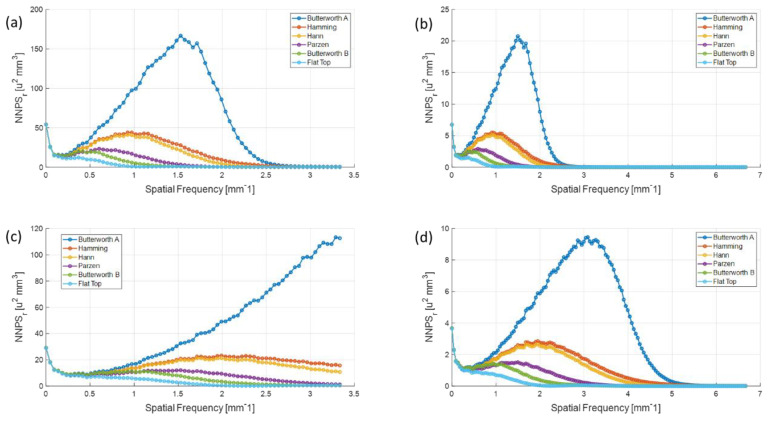
Radially averaged 1D NPS for each configuration from (**a**) (1, 1), (**b**) (1, 2), (**c**) (2, 1), and (**d**) (2, 2) with different filter schemes.

Figure 12 shows the radially averaged 1D NPS for each configuration with different filter schemes. The standard reconstructed image resolution (configuration (1, 1) and (2, 1)) gave higher noise properties compared to the high-resolution images (configuration (1, 2) and (2, 2)). We also observed that the peak of the 1D NNPSs from the higher detector resolution was at larger spatial frequencies, which demonstrates that the noise was distributed up to a higher frequency when the smaller pixels were used in the detector. The NPSs decreased as the intensity of high-frequency smoothing increased. 

## 4. Discussion

We herein designed band-limited filters for all schemes. These can effectively retrieve the sampled projections because the projections are discretized into each detector pixel so that it is band limited in the Fourier domain [24]. As a result, band-limited filters lead to the removal of unnecessary noise signals at high frequencies.

There is no universal filter in CT imaging; therefore, the user should select an optimal smoothing window to observe the detailed internal structure with a purpose. Selecting an optimal window function is often based on experience rather than theory because we do not have a high level of knowledge about whether the imaging object is lying under a low-, mid-, or high-frequency range [25]. Thus, comparing the initial imaging performance of different filters and choosing the best solution for one’s purpose is a good approach [25]. The most important factor when selecting the filter scheme is the manner in which the filter removes as many of the unnecessary components as possible in the frequency domain. In this experimental study, the signals near the edge of each material that we aimed to observe mostly lie in the low-frequency range, and show severe MTF distortion in the images applied with a high-pass filter, such as Butterworth A in Figure 9a. In contrast, the results in Figure 11f indicate that the flat-top window preserved the MTF up to a high frequency without an aliasing among the six filter schemes in our experiment. This is because the reconstructed images applied with the flat-top window not only resulted in uniform pixel values but also showed small oscillations (less noise) in both the target and background, as shown in the radial profiles in Figure 8b.

The flat-top window is used for cases in which a frequency component is required to be measured with great accuracy, e.g., a fixed-sine source [26]. Measuring the MTFs in the frequency domain could be interpreted as a discrimination of the signals spreading near the circular edge region. If a much larger signal difference exists between the target and the background, such as the tungsten edge, filter selection would not have been significant. However, we measured the MTFs for materials having no significant signal difference against the background material (low-contrast imaging); therefore, the amplitude accuracy was a key factor because the principal components in the Fourier domain were largely positioned in the low-frequency area [27]. 

The MTFs were preserved well at higher frequencies from the images reconstructed with a higher resolution. We observed that there was an MTF preservation loss up to 1.77 times by comparing the *f*_50_ between configurations (1, 1) and (2, 2) in Figure 10f and Figure 11f when using the same target material and detector resolution. Therefore, using a UHR imaging protocol rather than a standard imaging configuration is recommended to understand the fine sharpness of low-contrast material if the detector is available to be operated at a higher resolution. 

However, the high-level smoothing window is not recommended for standard resolution imaging configuration, as shown by the disagreement in the order of relative contrast in Figure 10. As shown in Figure 10, the flat-top window provided little difference in *f*_50_s for different materials even though there was a clear discrimination in UHR imaging protocol. This was because the flat-top window overly smoothed the low-contrast object in the standard imaging, whereas the smoothing was still effective in UHR mode. 

The trend of 1D NNPS in the configuration (2, 1) showed that the noise was distributed over all of the spatial frequencies. This demonstrates the back-projection from the high-resolution to small-image array would largely reduce the quantum noise and result in uniformly distributed noise.

The main drawback of this study is that all materials used to measure the MTFs had low contrast against the background PMMA intensity. This limits the study of higher-object-contrast materials such as bone and contrast-enhanced imaging. Our future study will be directed toward the effect of various filter setups on higher-object-contrast materials.

## 5. Conclusions

In summary, we observed the effect of filter schemes on several low-contrast materials using standard and UHR imaging protocols. Although UHR image acquisition requires a higher acquisition time and greater radiation exposure, we obtained spatial resolution up to 1.77 times higher than that of standard acquisition. In addition, the performance of UHR was affected by the FBP filter schemes, showing different *f*_50_ values and different noise patterns for different filters. Therefore, one should consider the optimal window function that can provide the best performance when observing the fine structure of the imaging object before UHR acquisition while comparing both the MTF and NPS.

## Figures and Tables

**Table 1:** Specifications of the imaging conditions.

Gantry	Sweep angle	0° to 360° with 1° step	
	Source-to-detector distance	1330 mm	
	Isocenter-to-detector distance	660 mm	
X-ray tube	Tube voltage	40–120 kVp	
	Tube current	10–500 mA	
	Exposure duration	16 ms	
FPD		Standard acquisition	UHR acquisition
	Image matrix	1024 × 768	2048 × 1536
	Pixel interval	0.388 mm	0.194 mm
	Framerate	7.5 fps	30 fps
	Readout time per view	~55 ms	~220 ms
	Total acquisition time	24 s	48 s
	Total entrance surface dose (ESD)	2.82 mGy	11.3 mGy
Reconstruction		Standard reconstruction	UHR reconstruction
	Image matrix	512 × 512	1024 × 1024
	Pixel interval	0.3 mm	0.15 mm

**Table 2:** Each configuration protocol with different resolution settings.

	Standard Reconstruction	UHR Reconstruction
Standard acquisition	Configuration (1, 1)	Configuration (1, 2)
UHR acquisition	Configuration (2, 1)	Configuration (2, 2)

**Table 3:** Material index and name of each cylinder embedded in the American Association of Physicists in Medicine (AAPM) phantom.

Material Index	Material Name (Density (g/cc))
M1	Polyethylene (0.95)
M2	Polystyrene (1.05)
M3	Nylon (1.10)
M4	Acrylic (1.19)
M5	Polycarbonate (1.20)
Background	PMMA * (1.18)

* Poly methyl methacrylate (PMMA).

**Table 4:** Description of each window that was implemented with the ramp filter.

Window Title	Equation
(a) Butterworth A ^1^	$1/\sqrt{1+{\left(\frac{\omega }{f_{c}}\right)}^{2p}}$ if
(b) Hanning	$0.5\left(1+\cos\left(\frac{2\pi \omega }{L}\right)\right)$ if
(c) Hamming	$0.54-0.46\cos\left(\frac{2\pi \omega }{L}\right)$ if
(d) Parzen	$\{\begin{array}{c} 1-6{\left(\frac{\left|\omega \right|}{L/2}\right)}^{2}+6{\left(\frac{\left|\omega \right|}{L/2}\right)}^{3}\\ 2{\left(1-\frac{\left|\omega \right|}{L/2}\right)}^{3} \end{array}\;\begin{array}{c} \mathrm{if}\;0\leq \left|\omega \right|\leq \left(L-1\right)/4\;\\ \mathrm{if}\;\left(L-1\right)/4\leq \left|\omega \right|\leq \left(L-1\right)/2 \end{array}$
(e) Butterworth B ^2^	$1/\sqrt{1+{\left(\frac{\omega }{f_{c}}\right)}^{2p}}$
(f) Flat Top	$0.21-0.41\cos\left(\frac{2\pi \omega }{L-1}\right)+0.27\cos\left(\frac{4\pi \omega }{L-1}\right)-0.08\cos\left(\frac{6\pi \omega }{L-1}\right)+0.006\cos\left(\frac{8\pi \omega }{L-1}\right)$

^1^*p* = 6, fc = 0.4, ^2^ *p* = 2, fc = 0.15.
